# Emerging roles of long non-coding RNAs in osteoarthritis: from molecular mechanisms to therapeutic opportunities

**DOI:** 10.1080/15476286.2025.2585219

**Published:** 2025-11-04

**Authors:** Yiming Zhao, Yanyan Zhang, Yuan Peng, Zheng Zheng, Qijing Li, Jiefei Shen, Hang Wang, Fei Liu

**Affiliations:** a State Key Laboratory of Oral Diseases & National Center for Stomatology & National Clinical Research Center for Oral Diseases & West China School of Stomatology, Sichuan University, Chengdu, China; b Department of Prosthodontics, West China Hospital of Stomatology, Sichuan University, Chengdu, China

**Keywords:** Long non-coding RNAs, osteoarthritis, chondrocytes apoptosis, extracellular matrix degradation, inflammation, molecular mechanisms

## Abstract

Long non-coding RNAs (lncRNAs) exert a significant influence on the occurrence and progression of osteoarthritis (OA). LncRNAs are characterized by their multifunctional nature, capable of regulating the expression, transcription, translation, and structural function of target genes through various mechanisms, spanning epigenetic, transcriptional, post-transcriptional, and post-translational levels. This review examines the mechanisms and functions of lncRNAs in cell proliferation, differentiation, apoptosis, extracellular matrix (ECM) degradation, and inflammatory responses in chondrocytes, synovial cells, and mesenchymal stem cells (MSCs) from mice and humans associated with OA. We emphasize the integral role of lncRNAs in the OA disease process. Conclusively, we present insights into OA treatment from the perspective of targeting lncRNAs, addressing future development prospects and potential clinical applications.

## 1. Introduction

**Figure 1.**
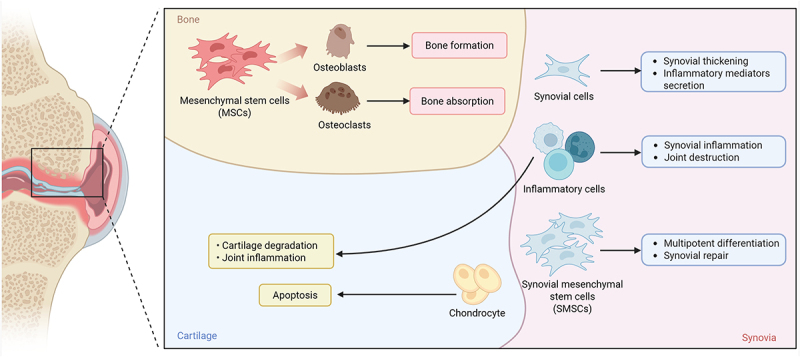
Cytological pathological process of OA. MSCs differentiate into osteoblasts and osteoclasts, contributing to bone formation and absorption. Synovial cells secrete inflammatory mediators, leading to synovial thickening and joint inflammation. Inflammatory cells further exacerbate joint destruction. SMSCs can undergo multipotent differentiation and participate in synovial repair. Chondrocytes undergo apoptosis, resulting in cartilage degradation and joint inflammation.

Osteoarthritis (OA) is a prevalent degenerative joint disease characterized by the progressive degradation of articular cartilage, subchondral bone remodelling, synovial inflammation, and osteophyte formation [1]. The pathogenesis of OA is multifactorial, involving a complex interplay of mechanical stress, inflammatory signalling, metabolic imbalance, and genetic predisposition [2–5]. Key cellular participants in OA include chondrocytes, synovial cells, and mesenchymal stem cells (MSCs) [6]. Cartilage degeneration is marked by the disruption of chondrocyte metabolic homoeostasis and the subsequent breakdown of the extracellular matrix (ECM) [7,8]. In OA, chondrocytes are stimulated by mechanical damage, inflammatory mediators, or metabolic defects, leading to phenotypic changes and the production of excessive degradative enzymes and inflammatory factors, further exacerbating cartilage destruction [9]. Synovial inflammation also plays a critical role in the pathophysiology of OA, with inflammation triggering the activation of synovial cells, which release pro-inflammatory cytokines and chemokines, further aggravating cartilage damage [10,11]. MSCs are endowed with multi-directional differentiation capacity, which allows them to differentiate into chondrocytes, osteoblasts, etc [12]. This characteristic confers upon them the potential to directly participate in the repair of damaged cartilage, making them a promising candidate for the treatment of OA [13]. The interplay between these cellular components and the progression of OA is further detailed in Figure 1, which provides a schematic representation of the complex interactions involved in joint pathogenesis.

Long non-coding RNAs (lncRNAs), a class of transcripts longer than 200 nucleotides that lack protein-coding capacity, have emerged as significant regulators of gene expression across multiple biological processes [14]. They are transcribed by RNA polymerase II and undergo splicing, 5’ capping, and 3’ polyadenylation, yet they exhibit low coding potential, high tissue specificity, and limited evolutionary conservation [15]. Since their initial discovery, lncRNAs have been implicated in diverse physiological and pathological processes due to their ability to interact with DNA, RNA, and proteins [16–19]. At the epigenetic level, lncRNAs can interact with DNA methyltransferases or recruit chromatin-modifying complexes to specific genomic loci, influencing DNA methylation patterns and histone modifications [20]. At the transcriptional level, lncRNAs can regulate the activity of transcription factors and RNA polymerase complexes [21]. Post-transcriptionally, lncRNAs can act as miRNA sponges or affect mRNA stability, splicing and translation [22,23]. Finally, at the post-translational level, lncRNAs can modulate protein stability, localization and function by interacting with protein complexes or regulating protein degradation pathways [24]. These multifaceted regulatory functions endow lncRNAs with the capacity to orchestrate complex molecular networks relevant to disease progression. Under normal physiological conditions, they are involved in X-chromosome inactivation [25], chromatin organization [26], genomic imprinting [27], stem cell differentiation [28], immune homoeostasis [29], and tissue development [30,31]. In pathological contexts, dysregulation of lncRNAs has been linked to the pathogenesis of a wide spectrum of diseases, including cancers [32], cardiovascular disorders [33], autoimmune diseases [34], and degenerative joint diseases such as OA [35].

Recent studies have increasingly linked lncRNA dysregulation to the initiation and progression of OA, particularly in mediating chondrocyte apoptosis, ECM degradation, inflammatory responses, and osteogenic differentiation [36–39]. Several lncRNAs, such as HOTAIR, H19, MEG3, and MALAT1, have been identified to either promote disease progression or facilitate cartilage repair through regulation of signalling pathways such as Wnt/β-catenin, NF-κB, and MAPK [40]. For instance, HOTAIR enhances cartilage degradation via epigenetic silencing of target genes [41], while MEG3 appears to exert protective effects by inhibiting inflammatory mediators [42,43]. These findings suggest that lncRNAs not only act as biomarkers reflective of OA clinical characteristics, but may also represent novel therapeutic targets for disease modification.

While prior reviews have explored the roles of individual lncRNAs in OA, few have provided a comprehensive synthesis of how lncRNAs function across multiple regulatory levels in OA. The innovation of this review lies in its systematic dissection of lncRNA-mediated mechanisms at four regulatory tiers – epigenetic, transcriptional, post-transcriptional, and post-translational – within the framework of OA pathogenesis. By consolidating current findings, we aim to elucidate how lncRNAs coordinate molecular events involved in cartilage degradation, inflammation, and tissue repair, and to discuss their implications in OA diagnostics and therapeutics.

**Figure 2.**
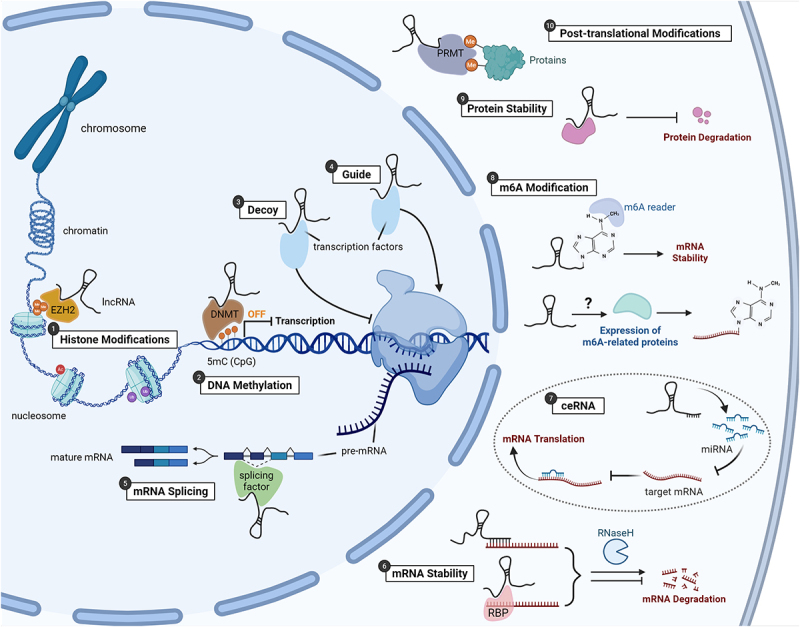
Integration of lncRNAs’ role in OA pathophysiology in this review. The intricate regulatory roles of lncRNAs in OA pathophysiology are highlighted, showing how they modulate various gene expression and cellular processes critical to OA. These include histone modifications, DNA methylation, transcription factor interactions – acting as decoys or guides, mRNA splicing, mRNA stability regulation, the competitive endogenous RNA (ceRNA) mechanism that influences miRNA activity, mRNA modifications such as N6-methyladenosine (m6A) that affect stability and translation, modulation of protein stability, and post-translational modifications.

To comprehensively explore the role of lncRNAs in OA, we conducted a systematic literature search across multiple databases, including PubMed, Scopus, Web of Science, and Google Scholar, covering the period from January 2010 to January 2025. The search strategy employed the following Boolean operators and keywords: (‘long non-coding RNA’ OR ‘lncRNA’) AND (‘osteoarthritis’ OR ‘OA’) AND (‘mechanism’ OR ‘pathogenesis’ OR ‘regulation’ OR ‘function’). As depicted in Figure 2, which outlines the multilayered roles of lncRNAs in OA, our review provides a critical overview of their biological functions, regulatory mechanisms, and therapeutic relevance, with the goal of informing future research and clinical translation in OA.

## 2. LncRNAs in OA: regulation at the epigenetic modification level

Epigenetic regulation is a crucial layer of gene expression control that does not alter the underlying DNA sequence but modulates chromatin structure and gene accessibility through mechanisms such as DNA methylation, histone modifications, and chromatin remodelling [44]. In recent years, lncRNAs have emerged as key regulators of these epigenetic processes [45]. In OA, accumulating evidence indicates that lncRNAs play an essential role in modulating the epigenetic machinery of chondrocytes and other joint-resident cells [46]. Through the recruitment or inhibition of DNA methyltransferases (DNMTs), coordination with histone-modifying enzymes such as EZH2, p300, or SIRT1, and modulation of chromatin accessibility, lncRNAs contribute to the dysregulation of gene expression networks that drive cartilage degradation, inflammation, and impaired chondrogenesis. Understanding the epigenetic functions of lncRNAs in OA not only elucidates disease mechanisms but also offers promising therapeutic avenues by targeting these non-coding regulators to restore epigenetic balance and joint homoeostasis.

### 2.1. DNA methylation

DNA methylation is one of the most important epigenetic mechanisms to regulate gene expression, which is highly dynamic during development and specifically maintained in somatic cells, typically associated with the repression of gene expression [47]. The methylation of cytosine residues at the C5 position within cytosine-phospho-guanosine (CpG) dinucleotides has long been recognized as a key epigenetic silencing mechanism. In humans, the majority of DNA methylation occurs in CpG-rich regions known as CpG islands, which are often located within gene promoters and predominantly remain unmethylated under normal conditions [48].

**Figure 3.**
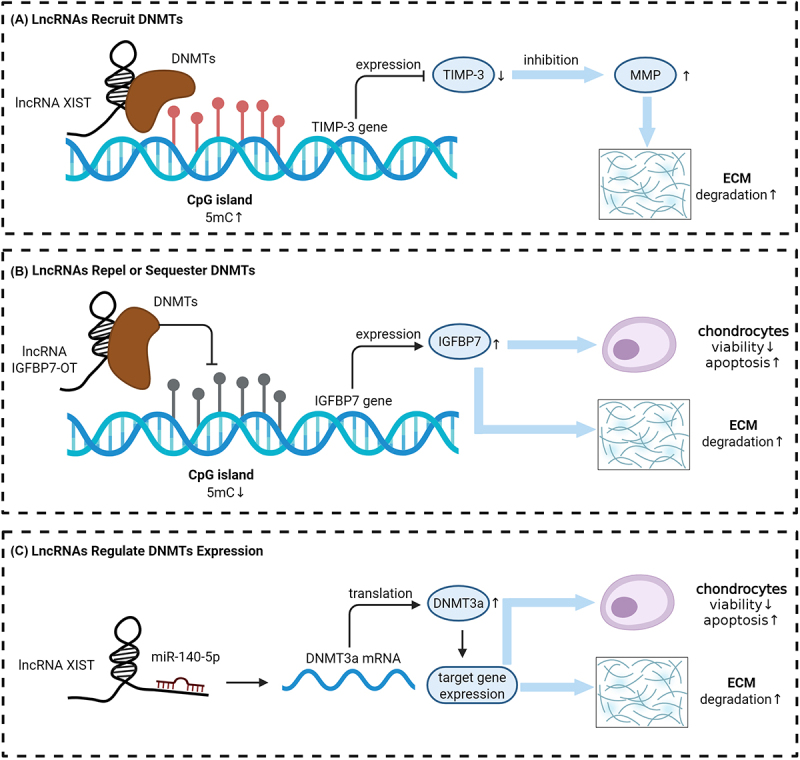
LncRNA-Mediated DNA methylation in OA. (a) LncRNAs recruit DNMTs: lncRNA xist binds to DNMTs, increasing 5mC at CpG islands and repressing TIMP-3 expression, leading to enhanced mmp activity and ecm degradation. (b) LncRNAs repel or sequester DNMTs: lncRNA IGFBP7-OT prevents DNMT binding, reducing 5mC and inducing IGFBP7 expression, promoting chondrocyte viability, reducing apoptosis, and increasing ecm degradation. (c) LncRNAs regulate DNMTs expression: lncRNA XIST interacts with miR-140-5p to upregulate DNMT3a translation, enhancing target gene expression and affecting chondrocyte viability, apoptosis, and ECM degradation.

DNA methylation is catalysed by DNMTs, which transfer a methyl group onto the C5 position of a cytosine within CpG dinucleotides to form 5-methylcytosine (5mC). DNMT1, often referred to as the maintenance DNMT, preserves existing methylation patterns during DNA replication, while DNMT3a and DNMT3b are involved in *de novo* methylation [49]. The Ten-Eleven Translocation enzyme family antagonizes the function of the DNMT family by oxidizing 5mC, leading to active DNA demethylation [50]. As depicted in Figure 3, during the pathophysiological process of OA, lncRNAs can participate in DNA methylation by recruiting, repelling, or modulating the expression of DNMTs, thereby shaping gene expression patterns. While lncRNA-mediated DNA demethylation has been described in other diseases [51], such mechanisms have not yet been reported in OA-related research, to the best of our knowledge.

#### 2.1.1. LncRNAs recruit DNMTs

The recruitment of DNMTs by lncRNAs constitutes a crucial mechanism for DNA methylation regulation in OA. LncRNAs specifically bind to DNMTs, directing them to the promoters of target genes, thereby altering their methylation patterns and transcriptional activity.

For example, lncRNA XIST is overexpressed in OA cartilage and IL-1β-treated chondrocytes, contributing to OA progression by interacting with DNMT1, DNMT3a, and DNMT3b. Chen et al. demonstrated that XIST recruits these DNMTs to the TIMP-3 promoter, leading to its hypermethylation and transcriptional suppression [52]. TIMP-3 is a critical inhibitor of matrix metalloproteinases (MMPs) that degrade collagen in OA chondrocytes. Overexpression of XIST increases DNMTs enrichment at the TIMP-3 promoter, while silencing XIST decreases methylation, upregulates TIMP-3 expression, and reduces collagen degradation. These findings underline the therapeutic potential of targeting XIST to regulate methylation and restore cartilage integrity.

In support of the broader relevance of lncRNA-DNMT interactions in musculoskeletal disorders, a study by Li et al. demonstrated that lncRNA SNHG1 regulates DNA methylation during bone marrow mesenchymal stem cell (BMSC) differentiation in an osteoporosis model [53]. SNHG1 interacts with PTBP1, facilitating the recruitment of DNMT1 to the osteoprotegerin (OPG) promoter, resulting in its hypermethylation and transcriptional repression. Since OPG is a key regulator of bone metabolism, its downregulation alters the osteogenic-adipogenic balance of BMSCs, contributing to osteoporosis pathogenesis. Although conducted in a different disease context, these findings highlight a conserved epigenetic mechanism by which lncRNAs guide DNMTs to specific gene loci, reinforcing the idea that lncRNA-mediated DNMT recruitment may not be disease-specific but may function differently depending on the pathological context of each skeletal disorder.

#### 2.1.2. LncRNAs repel or sequester DNMTs

Although current studies have not demonstrated that lncRNAs participate in demethylation by cooperating with demethylation-related enzymes in OA, some lncRNAs have been shown to negatively regulate DNA methylation by excluding or sequestering DNMTs, thereby facilitating gene expression.

The study by Tang et al. provides an example of this mechanism in action. They discovered that lncRNA IGFBP7-OT is upregulated in OA cartilage, and its interaction with DNMT1 and DNMT3a results in decreased binding to the IGFBP7 promoter. This interaction leads to promoter hypomethylation and subsequent upregulation of IGFBP7 expression [36]. IGFBP7 is a multifunctional regulatory protein that modulates cell proliferation, apoptosis, tissue development and repair, tumour suppression, and metabolic processes by inhibiting the IGF signalling pathway. Elevated IGFBP7 levels impair chondrocyte viability, promote apoptosis, and reduce ECM synthesis, thus exacerbating OA progression.

Similarly, lncRNA HOTAIRM1 has been shown to reduce DNMT1 expression and binding to the HOXA2 promoter in dental follicle stem cells, leading to its hypomethylation and enhanced osteogenic differentiation [54]. HOTAIRM1 directly interacts with CpG islands at the HOXA2 promoter, promoting gene expression by preventing DNMT1-mediated silencing. This finding supports the broader relevance of lncRNA-mediated DNMT exclusion in skeletal tissue remodelling.

#### 2.1.3. LncRNAs regulate DNMTs expression

LncRNAs also regulate DNMTs expression through interaction with miRNAs, thereby modulating the methylation status of target genes and influencing the progression of OA. miRNAs can bind to 3’ untranslated regions (UTR) of downstream target genes to degrade mRNAs or inhibit translation, further regulating the progression of diseases.

The study reveals that XIST interacts with miR-149-5p, a miRNA that targets DNMT3a [55]. By sponging miR-149-5p, XIST alleviates the inhibition of DNMT3a mRNA by miR-149-5p, thereby increasing DNMT3a expression. This increase in DNMT3a activity contributes to the methylation of DNA, particularly in the promoter regions of genes involved in OA pathogenesis. This process suppresses the viability of OA chondrocytes, promotes cell apoptosis, and accelerates the degradation of the ECM.

Beyond chondrocyte regulation, lncRNA-mediated control of DNMT3a via miRNAs has also been implicated in osteoclast biology. For instance, lncRNA MIR193BHG was shown to increase DNMT3a expression by inhibiting miR-489-3p, thereby enhancing osteoclast differentiation and bone resorption in the setting of breast cancer bone metastasis [56]. This regulatory interaction contributes to bone resorption and highlights the potential relevance of lncRNA-miRNA-DNMT3a signalling across distinct skeletal conditions.

### 2.2. Histone modifications

**Figure 4.**
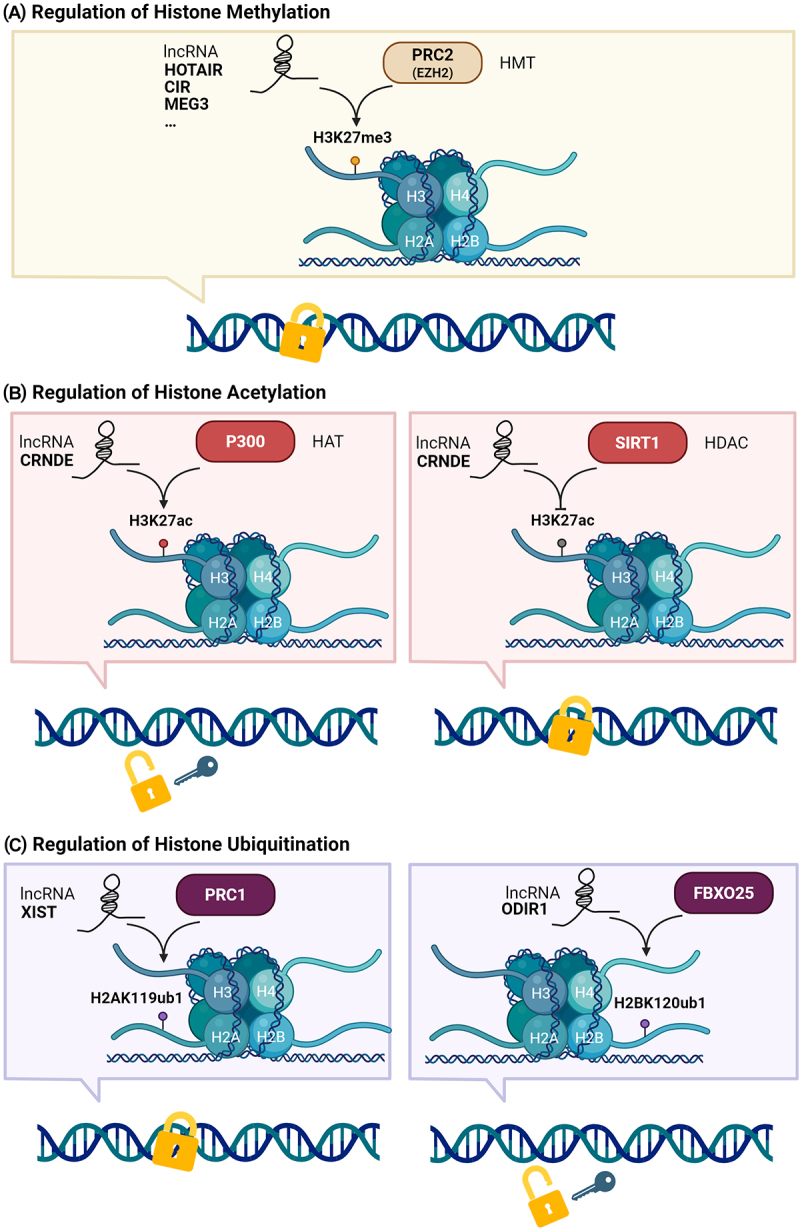
LncRNA-Mediated histone modifications in OA. (a) Histone methylation: lncRNAs HOTAIR, CIR, and MEG3 interact with PRC2 and EZH2, promoting H3K27me3, thereby influencing OA-related gene expression. (b) Histone acetylation: lncRNA CRNDE modulates H3K27ac levels via interactions with P300/HAT and SIRT1/HDAC, respectively, impacting OA pathogenesis. (c) Histone ubiquitination: lncRNA XIST mediates H2AK119ub1 via PRC1, while ODR1 promotes H2BK120ub1 via FBXO25, regulating chromatin structure in OA.

Histone modifications are a crucial component of epigenetics, regulating gene expression with precision by altering the structure and function of chromatin [57]. The primary types of histone modifications include methylation, acetylation, and ubiquitination. These modifications can activate or repress transcription, depending on the type of modification and its specific location on the histone. The core histones (H2A, H2B, H3, and H4) form nucleosomes, which are the basic units of chromatin. Histone modifications regulate gene accessibility and transcriptional activity by changing the structure of nucleosomes. LncRNAs can interact with histone-modifying enzymes, thereby influencing the patterns of histone modifications. This mechanism is particularly important in OA, where lncRNAs regulate histone modifications to affect chondrocyte function and cartilage homoeostasis. Figure 4 illustrates the mechanisms by which lncRNAs modulate histone modifications in OA, impacting chromatin structure and gene expression.

The Polycomb group (PcG) proteins play an essential role in the histone modification processes regulated by lncRNAs in the pathophysiology of OA, primarily through the mediation of histone modifications by the PRC1 and PRC2 complexes, thereby maintaining transcriptional repression of genes [58]. The PRC1 complex contains the E3 ubiquitin ligase subunit RING1a or RING1b, which catalyzes the monoubiquitination of histone H2A at lysine 119 (H2AK119ub1). This modification leads to chromatin compaction and subsequent gene silencing. The PRC2 complex contains the histone methyltransferase (HMT) subunit EZH1 or EZH2, which catalyzes the trimethylation of histone H3 at lysine 27 (H3K27me3), a modification also associated with gene silencing.

#### 2.2.1. Regulation of histone methylation

Histone methylation, mediated by HMTs, is crucial for chromatin condensation and transcriptional repression. This modification involves the addition of methyl groups to lysine or arginine residues on histones, particularly on the N-terminal tails of histone H3 and H4. In OA, lncRNAs primarily regulate histone methylation by interacting with chromatin-modifying complexes that possess HMTs activity, such as PRC2. Previous studies have indicated that approximately 24% of human lncRNAs can physically bind to EZH2 and recruit it to target genes [59].

LncRNA HOTAIR is highly expressed in OA chondrocytes and promotes disease progression by mediating histone methylation. HOTAIR binds to the PRC2 complex, inducing H3K27me3 in the WIF-1 promoter, which represses WIF-1 expression. This repression activates the Wnt/β-catenin pathway, increasing catabolic gene expression and promoting cartilage degradation [41]. This study provides the first evidence linking HOTAIR to OA progression through histone methylation, highlighting its potential as a therapeutic target.

Both lncRNA CIR and MEG3 interact with EZH2 to mediate histone H3K27me3, regulating chondrogenesis. CIR is upregulated in OA [60,61], binding to EZH2 to suppress ATOH8 expression via H3K27me3, thus inhibiting chondrogenesis [62]. Similarly, MEG3 binds to EZH2 to induce H3K27me3, repressing TRIB2 expression and impairing chondrogenesis [63]. These findings suggest that targeting lncRNA CIR and MEG3 could promote chondrogenesis and serve as potential therapeutic strategies for OA.

#### 2.2.2. Regulation of histone acetylation

Histone acetylation, particularly on lysine residues of histone proteins, is linked to transcriptional activation and is mediated by histone acetyltransferases (HATs), while being repressed by histone deacetylases (HDACs). LncRNAs interact with various chromatin modifiers, including HATs and HDACs, to regulate the acetylation status of histones, thereby affecting gene expression programs in a cell type-specific manner. For example, p300/CBP, a well-known HAT, can interact with lncRNAs to promote histone acetylation and gene activation. Conversely, SIRT1, an NAD^+^ -dependent HDAC, can deacetylate histones, leading to transcriptional repression and regulation of cellular processes such as metabolism and apoptosis.

LncRNA CRNDE has been shown to regulate histone acetylation in OA through interactions with both p300 and SIRT1. In one study, CRNDE promoted the recruitment of p300 to the DACT1 promoter, increasing the acetylation of histone H3 at lysine 27 (H3K27ac) and enhancing DACT1 expression, which inhibited the Wnt/β-catenin pathway and mitigated OA progression [64]. In another study, CRNDE interacted with SIRT1 to reduce H3K27ac and regulate SOX9 expression, thereby promoting chondrogenic differentiation and cartilage repair [39]. These findings demonstrate that CRNDE modulates histone acetylation via both HATs and HDACs, highlighting its potential as a therapeutic target for OA.

#### 2.2.3. Regulation of histone ubiquitination

Histone ubiquitination plays a key role in modulating chromatin structure and gene expression, with distinct outcomes depending on the histone targeted. Monoubiquitination of H2A leads to a more condensed chromatin structure, repressing gene expression, while H2B monoubiquitination results in a more open chromatin structure, facilitating gene expression.

In OA, lncRNAs have been shown to regulate histone ubiquitination with significant implications for disease progression and cartilage repair. For instance, lncRNA Xist recruits PcG proteins to facilitate H2AK119ub1, contributing to chromatin silencing and gene repression. This process is mediated through the Xist RNA polycomb interaction domain XR-PID, which interacts with the PCGF3/5-PRC1 complex via the RNA-binding protein hnRNPK [65]. Conversely, lncRNA ODIR1 in hUC-MSCs promotes osteogenic differentiation by facilitating the degradation of FBXO25, a protein that increases the monoubiquitination of histone H2B at lysine 120 (H2BK120ub1) and H3K4me3, both of which promote an open chromatin structure and enhance the expression of key osteoblast markers [66]. These findings underscore the dual role of lncRNAs in modulating histone ubiquitination, either repressing or activating gene expression through interactions with specific chromatin modifiers. This regulatory mechanism highlights the potential of lncRNAs as therapeutic targets for OA, offering new insights into disease pathogenesis and treatment strategies.

## 3. LncRNAs in OA: regulation at the transcription level

LncRNAs are emerging as important transcription regulators of diverse biological functions. Studies in the past decade indicate that a large number of lncRNAs are enriched in the nucleus and originate from transcriptionally active regulatory elements. These lncRNAs associate with transcription factors and chromatin regulatory elements to fine-tune the transcriptional output of protein coding genes. Transcription factors are sequence-specific DNA binding proteins that can activate or repress transcription. In the pathogenesis of OA, lncRNAs may act as decoys or guides to modulate gene expression at the transcriptional level.

### 3.1. LncRNA-Mediated decoy function in transcriptional regulation

LncRNAs can function as decoys to competitively bind transcription factors or other regulatory molecules, thereby preventing these proteins from interacting with their target DNA sequences. This competitive binding effectively sequesters the regulatory proteins, leading to the changes of gene transcription. A typical example is the enrichment of lncRNA HOXA11-AS expression in the nucleus, indicating its potential role in the transcriptional regulation of gene expression. It has been confirmed that HOXA11-AS regulates metabolic homoeostasis and ensures the integrity of chondrocytes. Research has demonstrated HOXA11-AS acts as a decoy to sequester the transcriptional repressor POU2F2, thereby relieving its inhibitory effect on SLC3A2 expression. SLC3A2, a key regulator of ferroptosis, is upregulated by HOXA11-AS, effectively inhibiting chondrocyte ferroptosis and mitigating OA progression [67]. This mechanism underscores the decoy function of lncRNAs in transcriptional regulation, presenting a novel therapeutic strategy for OA.

Similar decoy functions have been identified in other disease models. In ovarian cancer, lncRNA GAS5 binds to the transcription factor CEBPB, preventing its activation of GDF15 transcription, thereby reducing cell viability and promoting apoptosis [68]. In gastric cancer, lncRNA BC002811 promotes metastasis by binding and blocking SOX2, which in turn represses PTEN transcription [69]. These findings support the broader role of lncRNA-mediated decoy mechanisms in transcriptional regulation across pathological contexts.

### 3.2. LncRNA-Driven guide function in transcriptional targeting

LncRNAs serve as molecular guides to direct ribonucleoprotein complexes to specific genomic loci. These lncRNAs bind to target effector proteins and facilitate the recruitment of these proteins to particular DNA sequences, thereby influencing gene transcription in a sequence-specific manner. This targeted recruitment can result in either activation or repression of transcription, depending on the nature of the recruited proteins. The guide function of lncRNAs enables precise control over transcriptional programs by facilitating the spatial and temporal targeting of regulatory complexes. For example, lncRNA H19, which is highly expressed in OA, exerts protective effects against inflammatory responses and chondrocyte apoptosis by serving as a molecular guide to recruit TP53, thereby upregulating IL-38 and activating the IL-36 R. Specifically, H19 binds to TP53, a transcription factor, promoting IL-38 expression by facilitating TP53 binding to the IL-38 promoter. This interaction between H19 and TP53 exemplifies the guide function of lncRNAs, where H19 sequesters TP53 to specific genomic loci, enabling precise control over transcriptional programs [70]. The subsequent interaction between IL-38 and IL-36 R further enhances the protective effects, highlighting the role of lncRNA H19 and TP53 in modulating OA progression.

Another example involves the lncRNA HOTTIP, which promotes osteogenic differentiation of BMSCs by interacting with WDR5, a component of transcriptional regulatory complexes, and guiding its recruitment to the β-catenin promoter [71]. This enhances β-catenin transcription and activates Wnt/β-catenin signalling.

## 4. LncRNAs in OA: regulation at the post-transcription level

### 4.1. LncRNA regulation of mRNA splicing

RNA splicing is a process that removes introns and joins exons in pre-mRNA, leading to mature mRNA that can be translated into proteins. LncRNAs can influence alternative splicing by interacting with splicing factors, thereby affecting the diversity of protein isoforms produced from a single gene. Serine/arginine-rich (SR) proteins and heterogeneous nuclear ribonucleoproteins (HnRNPs) are the two primary families of alternative splicing factors that have been extensively studied [72,73]. SR proteins are a family of RNA-binding proteins and regulate both general and alternative splicing. SRSF3 is a quintessential member of the serine/arginine-rich protein family. In temporomandibular joint OA, lncRNA EPS is downregulated in condylar tissues and negatively correlates with inflammatory factors. SRSF3 is a splicer of PKM that induces primary PKM RNA transcripts switching to PKM2 [74]. EPS competitively binds to SRSF3, inhibiting PKM2 formation and subsequent activation of the PKM2/NF-κB pathway [38]. This interaction reduces the expression of inflammatory factors. The absence of EPS promotes inflammation in condylar chondrocytes, while its restoration exhibits anti-inflammatory effects. Thus, EPS plays an essential role in modulating inflammation in temporomandibular joint OA by regulating PKM2 expression.

HnRNPs are special nucleic acid binding proteins that create complexes with heterogeneous nuclear RNA (HnRNA). HnRNPLL is a member of the HnRNPs family, which plays a crucial role in modulating alternative splicing and gene expression regulation. Studies have highlighted the distinct roles of lnc-PPP2R1B and its target mRNA PPP2R1B in osteogenesis. Lnc-PPP2R1B, a non-coding RNA, is up-regulated during the osteogenic differentiation of MSCs, while PPP2R1B is a coding mRNA that serves as a regulatory subunit of PP2A. Lnc-PPP2R1B promotes osteogenesis by interacting with HnRNPLL, to retain exon 2 and 3 in PPP2R1B transcripts, generating the functional isoform that maintains PP2A activity [75]. This interaction is crucial for activating the Wnt/β-catenin pathway, enhancing Runx2 and OSX expression, and ultimately promoting osteogenesis. This mechanism underscores the pivotal role of lncRNA-mediated alternative splicing in modulating gene expression and osteogenesis, providing insights into potential therapeutic targets for OA treatment.

Previous studies have assessed whole-genome differential splicing in OA and identified splice variation in 209 genes, which are enriched for terms related to the ECM, proteoglycans, and integrin surface interactions [76]. For genes such as ABI3BP, AKT1S1, and TPRM4, researchers have detected differences in the usage of splicing isoforms. These genes encode proteins associated with ECM proteins, negative regulators of the mTOR pathway, and transient receptor potential channels, respectively. However, the specific molecular mechanisms by which lncRNAs directly participate in mRNA splicing may require further experimental research to elucidate the precise roles of these lncRNAs in mRNA splicing.

### 4.2. LncRNA modulation of mRNA stability

mRNA stability is a critical factor in determining gene expression levels. The stability of mRNA is related to the integrity of its structure, which includes the 5’ cap, the 5’ UTR, the open reading frame, the 3’ UTR and the polyadenylated tail (Poly A tail) at the 3’ end [77,78]. The 5’ cap and Poly A tail are crucial for maintaining mRNA stability. When the 5’ cap and Poly A tail are present, mRNA is protected from degradation by exonucleases and can continue to be translated into proteins. However, when they are removed, mRNA is degraded by exonucleases, leading to a reduction in the synthesis of its encoded protein products [79,80]. LncRNAs play a pivotal role in the regulation of OA by directly targeting mRNA stability, a mechanism that operates independently of gene promoter or transcriptional level alterations. For example, lncRNA HOTAIR does not significantly affect the promoter activity of the ADAMTS-5 gene but markedly enhances the stability of ADAMTS-5 mRNA in OA chondrocytes, thereby promoting the degradation of the ECM [81]. LncRNAs can modulate mRNA stability either by directly binding to them or through interactions with RNA-binding proteins (RBPs).

LncRNAs can directly interact with mRNAs to modulate their stability, thereby regulating gene expression at the post-transcriptional level. For instance, lncRNA DANCR has been shown to directly bind to myc, STAT3, and Smad3 mRNAs, which play crucial roles in cell proliferation and chondrogenic differentiation of mesenchymal stem cells derived from synovium (SMSCs) [82]. Overexpression of DANCR significantly increased the mRNA and protein levels of myc, STAT3, and Smad3, while its knockdown had the opposite effect. Additionally, DANCR elongated the half-life of these mRNAs, indicating its role in stabilizing them. Using BLAST, regions of high complementarity between DANCR and these mRNAs were identified, and RIP-qPCR confirmed the direct interaction of DANCR with myc, STAT3, and Smad3 mRNAs. In OA, myc is essential for SMSC proliferation, while STAT3 and Smad3 are key regulators of chondrogenic differentiation. Thus, DANCR’s ability to stabilize these mRNAs directly contributes to OA progression by modulating the expression of critical genes involved in cartilage repair and maintenance.

RBPs are important regulatory molecules within cells that bind to RNA to regulate its splicing, transport, translation and degradation, thereby maintaining normal cellular physiological functions [83]. Previous studies have demonstrated that the lncRNA WDR11-AS1 is downregulated in cartilage tissues from OA patients, and to promote ECM synthesis in OA chondrocytes with knockdown and overexpression experiments. This function of WDR11-AS1 was linked to its ability to interact with PABPC1. PABPC1 was discovered to bind ECM-related mRNAs such as SOX9, and the inhibition of PABPC1 improved the mRNA stability of SOX9 to mitigate OA progression [37]. In addition, the expression of lncRNA OIP5-AS1 was significantly reduced in the cartilage tissue of OA patients. The study found that OIP5-AS1 played a protective role in LPS-stimulated rat chondrocytes. OIP5-AS1 induced the stability and expression of PPAR-γ mRNA by recruiting RBP FUS, and then induced mitochondrial autophagy and inhibited inflammation by activating the AMPK/Akt/mTOR pathway [84].

In addition to lncRNAs that enhance mRNA stability, some lncRNAs can reduce mRNA stability, thereby promoting the progression of OA. A novel biomarker lncRNA AC006064.4–201 has been identified in chondrocytes, which is downregulated in aged and degenerated human cartilage. AC006064.4–201 can inhibit the binding of CDKN1B mRNA to PTBP1, thereby reducing the mRNA stability and consequently decreasing the protein synthesis of CDKN1B [85]. CDKN1B is a key regulator of cell cycle progression and is considered an important ageing marker in age-related diseases. Studies have found a positive correlation between CDKN1B and chondrocyte senescence as well as OA progression.

### 4.3. LncRNA mediation in ceRNA mechanism

The ceRNA mechanism refers to the competitive binding of miRNAs by lncRNAs that share miRNA response elements with mRNAs, thereby alleviating miRNA-mediated repression and enhancing the translational efficiency of mRNAs. In OA, this mechanism plays a pivotal role in the post-transcriptional regulation of genes involved in chondrocyte biology, inflammation, and ECM homoeostasis. Our comprehensive literature search has identified 39 uniformly upregulated and 23 uniformly downregulated lncRNAs implicated in the ceRNA mechanism in OA, as summarized in Table S1. Additionally, we have presented 5 inconsistently regulated lncRNAs, including NEAT1, MALAT1, PART-1, MCM3AP-AS1 and KCNQ1OT1, and their associated ceRNA networks in Table S2.

These lncRNAs exhibit variable expression patterns across different studies. Specifically, certain lncRNAs are found to promote inflammation, enhance apoptosis, and inhibit cell proliferation, which are detrimental processes in the joint. For instance, lncRNA HOTAIR has been demonstrated to modulate inflammation and apoptosis in chondrocytes, and its upregulation is associated with the promotion of cartilage degradation. It can act through the ceRNA mechanism by interacting with miR-222-3p [86], miR-17-5p [87], miR-20b [88], miR-107 [89], and miR-1277-5p [90] to facilitate the expression of target genes such as ADAM10, FUT2, and CXCL12, thereby participating in the pathogenesis of OA. Conversely, a subset of lncRNAs has been shown to exhibit protective effects on cells under stress, potentially mitigating the deleterious effects of OA. For example, lncRNA DANCR functions as a ceRNA by binding to miR-216a-5p, which in turn regulates chondrocyte apoptosis in OA [91]. As JAK2 is a direct target of miR-216a-5p, DANCR’s interaction with this miRNA modulates the JAK2/STAT3 signalling pathway in OA chondrocytes, subsequently enhancing chondrocyte proliferation and inhibiting apoptosis.

The majority of these lncRNAs exert their effects in chondrocytes, which are responsible for maintaining the integrity of articular cartilage. However, some lncRNAs also function in synovial cells, contributing to the inflammatory response within the joint. LncRNA NEAT1 has been found to be upregulated in synovial cells of osteoarthritis patients, where it binds to miR-181c, thereby promoting the expression of the OPN gene and enhancing the proliferation of synovial cells [92].

**Figure 5.**
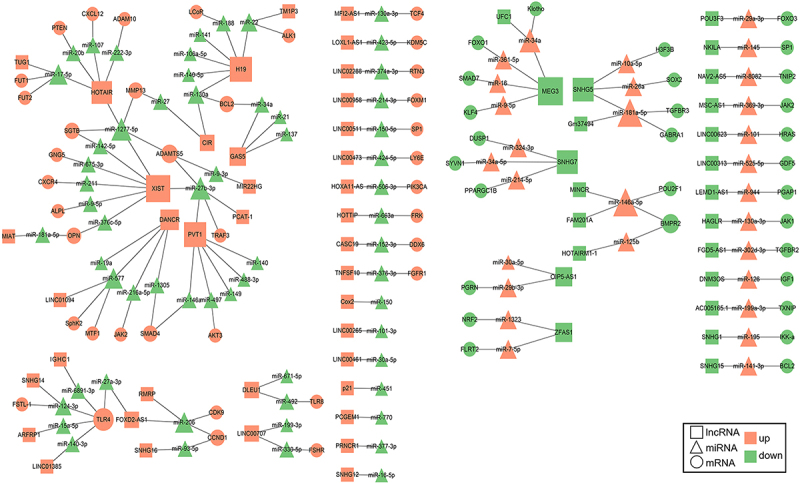
LncRNA-miRNA-mRNA ceRNA regulatory network in OA. The ceRNA regulatory network illustrated in this figure was generated through a systematic review of existing literature. Studies were identified by searching databases such as PubMed, web of Science, and Google Scholar using keywords related to ‘lncRNA-miRNA-mRNA ceRNA network’ and ‘OA ceRNA network’. Molecular interactions involving lncRNA, miRNA, and mRNA were extracted from these studies, including miRNA binding to lncRNA, miRNA regulation of mRNA, and competitive interactions between lncRNA and mRNA. These interactions were integrated to form the network, which was visualized using cytoscape.

We have constructed a lncRNA-miRNA-mRNA ceRNA regulatory network, as depicted in Figure 5. Analysis shows that TLR4 is a central hub in OA, where lncRNAs modulate a multitude of inflammatory responses and other pathophysiological processes through the ceRNA mechanism. In addition, we found that the ceRNA regulatory mechanisms of lncRNA in OA mainly involve the signalling pathways of MAPK/ERK, PI3K/AKT, Wnt/β-catenin, JAK/STAT, NF-κB, and TGF-β. The ceRNA mechanism involving lncRNAs in OA is multifaceted, with each lncRNA potentially interacting with multiple miRNAs and mRNAs, thereby influencing various biological processes. These interactions highlight the complexity of gene regulation in OA and suggest that targeting specific lncRNAs or miRNAs could provide therapeutic benefits.

Notably, lncRNA H19 also mitigates cartilage degeneration in developmental dysplasia of the hip by regulating the miR-483-5p/Dusp5 axis under mechanical stress, suggesting that ceRNA mechanisms involving lncRNAs are not exclusive to OA but extend to other cartilage-related disorders [93].

### 4.4. LncRNA control of mRNA modification

m6A is the most prevalent internal modification of mRNA, predominantly distributed in stop codons, 3’UTRs, precursor mRNA, coding sequence and inner long exon of matured mRNA. The m6A methylation process is dynamic and reversible, involving three crucial molecular compositions: m6A methyltransferases (writers), m6A demethylases (erasers) and m6A recognition factors (readers). These molecules are responsible for adding, removing, and recognizing m6A sites, respectively, and play crucial roles in normal biological processes and development. In OA, overexpression of the m6A methyltransferase METTL3 in chondrocytes treated with IL-1β reduces the levels of inflammatory cytokines, while IL-1β treatment can reverse the decrease in cytokines caused by METTL3 overexpression [94]. Moreover, silencing METTL3 in chondrocytes activates the NF-κB signalling pathway, thereby reducing IL-1β-induced apoptosis and inflammatory cytokine levels [95], indicating that m6A can modulate the progression of OA.

The mutual regulation between m6A and lncRNA predominantly involves two parts. On the one hand, m6A modification affects the RNA-protein and the RNA-RNA interactions. In OA tissues and IL-1β-induced primary chondrocytes, the lncRNA LINC00680 is upregulated. Silencing LINC00680 restores chondrocyte proliferation and inhibits the degradation of ECM. The m6A methyltransferase METTL3 binds to the m6A sites of LINC00680 to enhance its expression. LINC00680 positively regulates SIRT1 mRNA through the m6A-binding protein IGF2BP2, stabilizing SIRT1 mRNA [96]. Additionally, the expression of lncRNA HS3ST3B1-IT1 and its maternal gene HS3ST3B1 is downregulated and positively correlated in OA cartilage. HS3ST3B1-IT1 upregulates HS3ST3B1 expression by blocking ubiquitination-mediated degradation. ALKBH5-mediated m6A demethylation stabilizes HS3ST3B1-IT1 RNA [97]. The upregulation of HS3ST3B1-IT1 inhibits OA progression by increasing HS3ST3B1 expression, suggesting that the HS3ST3B1-IT1/HS3ST3B1 axis could be a potential therapeutic target for OA.

On the other hand, lncRNAs regulate the function and expression of m6A-related proteins. For instance, silencing the lncRNA LINRIS reduces the expression of the m6A reader IGF2BP2, thereby inhibiting its downstream effects and modulating the proliferation of colorectal cancer [98]. The lncRNA GAS5-AS1 interacts with ALKBH5 to regulate GAS5 expression, and m6A-mediated GAS5 RNA degradation is closely related to the YTHDF2-dependent pathway in cervical cancer [99]. The lncRNA LNC942 can directly recruit the m6A writer METTL14, regulating the expression of downstream targets through post-transcriptional m6A modification and participating in the development of breast cancer [100]. However, the mechanisms underlying this regulatory mode in OA remain to be elucidated.

## 5. LncRNAs in OA: regulation at the post-translational level

LncRNAs play a significant role in the pathogenesis of OA by modulating the post-translational modifications (PTMs) and stability of metabolic enzymes, transcription factors, and signalling pathway proteins. These regulatory mechanisms directly impact the metabolic imbalance of chondrocytes, inflammatory responses, and degradation of the ECM.

### 5.1. Regulation of PTMs

PTMs are pivotal in dynamically regulating protein function, with lncRNAs playing a crucial role in modulating these modifications. In OA, lncRNAs intervene in key PTM processes such as methylation, phosphorylation, acetylation, and ubiquitination, thereby influencing the activity and localization of OA-related proteins. Among these PTMs, the methylation of arginine residues by protein arginine methyltransferases (PRMTs) is particularly significant. Type I PRMTs, including PRMT1, PRMT2, PRMT3, CARM1, PRMT6, and PRMT8, catalyse the formation of asymmetric dimethylarginine in target proteins, which is involved in a wide range of cellular processes.

A notable example is the interaction between lncRNA PILA and PRMT1 in human articular chondrocytes. PILA, which is highly upregulated in human OA cartilage, binds to PRMT1 and enhances its activity [101]. This interaction leads to the methylation of RNA helicase DHX9 by PRMT1, subsequently increasing the expression of TAK1. TAK1 encodes a kinase that promotes the activation of NF-κB signalling, a pathway critical for inflammation-induced extracellular matrix degradation in articular chondrocytes [101]. Thus, PILA not only modulates PRMT1 activity but also amplifies downstream inflammatory responses, highlighting its significant role in OA pathogenesis.

A similar regulatory mechanism has been reported in vascular smooth muscle cells, where lncRNA TUG1 facilitates the methylation of α-actin by interacting with the lysine methyltransferase EZH2 [102]. TUG1 forms a cytoplasmic complex with EZH2 and α-actin, and its knockdown disrupts this interaction and reduces α-actin methylation, thereby impairing F-actin polymerization. These findings demonstrate the involvement of lncRNAs in modulating methyltransferase activity and cytoskeletal dynamics through PTM regulation.

### 5.2. Regulation of protein stability

LncRNAs also influence the half-life and function of OA-related proteins by modulating their degradation or stabilization. LncRNA RP11-364P22.2, which is overexpressed in OA cartilage tissues and chondrocytes, exhibits high responsiveness to IL-1β stimulation [103]. This lncRNA binds to the transcription factor ATF3, stabilizing the protein and promoting its nuclear translocation. This process is crucial for IL-1β-induced growth inhibition, apoptosis, and the degradation of structural proteins, as well as the synthesis of the ECM degradation enzyme MMP-13 in chondrocytes [103]. The predicted binding site of RP11-364P22.2 on ATF3 is located in the 31–82 aa domain, which is not an active transcription binding domain. This interaction is essential for the activation of the IL-1β-induced NF-κB pathway. In chondrocytes lacking RP11-364P22.2, ATF3 stability is reduced, leading to accelerated degradation and a significant decrease in ATF3 protein levels. Consequently, the phosphorylation of IκB and p65 is attenuated, impairing the degradation of IκBα and thus inhibiting NF-κB activation [103].

## 6. Stage-specific roles of lncRNAs in the pathogenesis of OA

OA is a progressive disease characterized by stage-specific pathological processes, including early synovial inflammation, intermediate cartilage degradation, and late subchondral bone remodelling. While numerous studies have identified lncRNAs such as XIST, HOTAIR, CRNDE, and DANCR as key regulators in OA pathogenesis, most of these findings are based on analyses of advanced or unspecified disease stages.

Notably, some of these lncRNAs are involved in multiple aspects of OA pathology. For example, HOTAIR has been reported to promote inflammatory signalling and ECM degradation [86,90], while DANCR is associated with both cartilage destruction and osteogenic differentiation [82,91]. These observations imply that the same lncRNA may exert distinct or even opposite functions depending on the disease stage and cellular context.

However, current evidence remains fragmented, and there is a lack of systematic investigation into the temporal dynamics of lncRNA function during OA progression. Future studies incorporating longitudinal clinical samples, animal models with defined disease stages, and single-cell or time-series transcriptomics are essential to unravel the stage-dependent roles of lncRNAs and to optimize therapeutic targeting strategies.

## 7. Diagnostic and therapeutic potential of lncRNAs in OA

Recent evidence suggests that certain lncRNAs exhibit disease stage-specific expression patterns and strong tissue specificity, which endow them with value as early diagnostic biomarkers for OA. For instance, upregulation of HOTAIR and downregulation of MEG3 in early-stage OA cartilage have been proposed as indicators of disease onset [35]. Moreover, circulating lncRNAs detected in synovial fluid, plasma, or exosomes offer promising potential as non-invasive biomarkers for early diagnosis and prognosis [104]. Their dynamic expression patterns in response to joint stress, inflammation, and cartilage degeneration further support their application in monitoring disease progression and treatment response.

In addition to their diagnostic utility, lncRNAs are also emerging as novel druggable targets in OA. Several lncRNAs have been shown to regulate critical signalling pathways involved in OA pathogenesis, such as NF-κB, Wnt/β-catenin, PI3K/Akt, and TGF-β, making them attractive nodes for pharmacological intervention. For example, silencing HOTAIR has been found to reduce IL-1β-induced inflammatory responses and matrix degradation in chondrocytes [86], whereas overexpression of MEG3 can attenuate cartilage inflammation [42]. A concise summary of representative OA-related lncRNAs, including their regulatory mechanisms and potential clinical implications, is presented in Table 1.

**Table 1.** Representative lncRnas involved in OA pathogenesis and their potential clinical relevance.

LncRNA	Mechanism Highlighted in OA	Suggested Clinical Relevance*	Reference
HOTAIR	Promotes ECM degradation and inflammation	Potential biomarker and therapeutic target; upregulated in early OA cartilage	[41,81,86–90]
MEG3	Suppresses IL-1β-induced inflammation; inhibits matrix-degrading enzymes	Proposed early-stage biomarker; downregulated in OA	[42,43,63]
DANCR	Modulates chondrogenesis and cartilage matrix metabolism	May regulate cartilage repair/remodelling; possible target for intervention	[82,91]
CRNDE	Attenuates apoptosis, inflammation, and ECM degradation	Limited evidence; potential chondroprotective role	[39,64]
XIST	Regulates chondrocyte apoptosis and inflammatory gene expression	Upregulated in OA cartilage; under investigation for diagnostic value	[52,55,65]

*Note: Clinical relevance is based on preliminary evidence and has not been fully validated in patient cohorts.

The translation of lncRNA research into clinical applications for OA relies on the development of molecular tools capable of modulating pathogenic lncRNAs with precision. Targeting such lncRNAs through antisense oligonucleotides (ASOs), small interfering RNAs (siRNAs) or CRISPR-Cas systems may provide a new therapeutic strategy for OA management.

ASOs are short, single-stranded, chemically modified DNA or RNA oligonucleotides designed to hybridize sequence-specifically to complementary RNA transcripts [105]. Upon binding, ASOs elicit RNase H-mediated degradation of the target RNA or sterically block critical RNA-RNA or RNA-protein interactions, thereby suppressing transcript function [106]. For example, H19-directed ASOs have been loaded into magnetic metal-organic framework (MMOF) nanoparticles and administered intra-articularly to the knee in the destabilization of the medial meniscus mouse model to achieve site-specific, osteocyte-targeted delivery. Targeted inhibition of H19 using ASO-loaded MMOF markedly attenuated subchondral bone remodelling and ameliorated the OA phenotype [107].

siRNAs are short double-stranded RNA molecules, typically 21–25 nucleotides in length [108]. They exert their function by guiding the RNA-induced silencing complex to complementary target transcripts, leading to sequence-specific degradation [109]. By selectively eliminating cytoplasmic lncRNA species or functional transcript fragments, siRNA-mediated knockdown can abrogate downstream post-transcriptional regulatory networks. For instance, siRNA-mediated silencing of lncRNA CIR in chondrocytes has been shown to promote synthesis of type II collagen and aggrecan while concurrently downregulating expression of matrix-degrading enzymes such as MMP13 and ADAMTS5, thereby restoring ECM homoeostasis in OA models [110].

CRISPR/Cas-based genome editing has revolutionized molecular biology by enabling precise and efficient DNA manipulation. Within lncRNA research, CRISPR/Cas-based approaches allow targeted knockout, activation, or modulation of lncRNA loci at the DNA level, thereby providing durable changes in lncRNA expression and facilitating the study of causal relationships in disease progression [111]. Nevertheless, the complex spatial structures of lncRNAs and their frequent overlap with host or neighbouring genes present significant challenges, underscoring the need for further optimization of genome-editing strategies for therapeutic application.

Effective clinical translation also depends on advanced delivery strategies and combination approaches. Nanoparticles, hydrogels, and exosome-based carriers are being developed to improve stability, tissue targeting, and controlled release [112]. Furthermore, lncRNA-targeted therapies may be integrated with anti-inflammatory drugs, stem cell therapy, or tissue engineering, creating synergistic effects that promote cartilage repair and functional recovery rather than merely alleviating symptoms. Addressing challenges such as delivery efficiency, immunogenicity, long-term safety, and species-specific variability will be essential to realize the clinical potential of these emerging strategies.

## 8. Conclusion and perspectives

LncRNAs have emerged as critical regulators in the pathogenesis of OA, exerting control over gene expression and cellular processes through diverse mechanisms. They operate at multiple levels – including epigenetic, transcriptional, post-transcriptional, and post-translational regulation – by interacting with DNA methyltransferases, histone-modifying enzymes, transcription factors, splicing regulators, RNA-binding proteins, and m6A modification machinery. Mechanistically, lncRNAs function both in cis, influencing the expression of neighbouring genes by modulating chromatin architecture or transcriptional elongation, and in trans, regulating distant genes through interactions with protein partners or by engaging in ceRNA networks. Through these multifaceted actions, lncRNAs orchestrate key aspects of OA pathology, including chondrocyte metabolism, inflammation, extracellular matrix homoeostasis, and cartilage degradation. Representative examples such as XIST, HOTAIR, CRNDE, and DANCR underscore their significance as central players in OA progression, while also highlighting their potential as therapeutic targets and clinical biomarkers.

Despite significant advancements in understanding the roles of lncRNAs in OA, several challenges and limitations remain. One major issue is the species-specific differences observed between human and animal models, which can complicate the translation of findings from pre-clinical studies to clinical applications. For instance, the expression patterns and functional roles of certain lncRNAs may vary between species, necessitating careful validation in human tissues. Additionally, the complexity of lncRNA regulatory networks often make it difficult to delineate the precise molecular mechanisms involved in OA pathology. Outstanding questions include how lncRNAs coordinate crosstalk among signalling pathways and how their post-transcriptional modifications affect stability and function.

Future research should focus on addressing these challenges by leveraging advanced technologies such as single-cell RNA sequencing, spatial transcriptomics, multi-omics approaches, and CRISPR-based gene editing. These approaches will be instrumental in identifying novel lncRNAs with diagnostic, prognostic, and therapeutic potential, and in uncovering their context-specific regulatory roles. From a therapeutic standpoint, lncRNA-targeted therapies, including ASOs, siRNAs and nanoparticle-based delivery systems, are currently under preclinical exploration and hold considerable promise. Combining lncRNA-targeted therapies with existing treatments may offer synergistic benefits for cartilage repair and disease modification.

The clinical translation of lncRNA-based diagnostics and therapeutics requires overcoming several hurdles, including efficient delivery to joint tissues, minimizing off-target effects, and ensuring long-term safety. Nonetheless, the high tissue specificity, dynamic regulation, and functional versatility of lncRNAs make them uniquely suited for personalized medicine applications in OA. Establishing reliable lncRNA biomarker panels and developing targeted intervention strategies could significantly improve disease management, patient stratification, and therapeutic efficacy.

In conclusion, lncRNAs represent a promising frontier in OA research and therapy. Their multifaceted regulatory roles in OA pathogenesis provide new insights into disease mechanisms and open avenues for innovative therapeutic strategies. As our understanding of lncRNA biology continues to expand, targeting these molecules may pave the way for more effective and personalized treatments for OA, ultimately improving patient outcomes and quality of life. The successful translation of lncRNA research into clinical applications will not only advance our understanding of OA but also contribute to the broader field of non-coding RNA biology.

## Data Availability

The supplementary tables supporting this study have been deposited in Zenodo and are publicly available (https://doi.org/10.5281/zenodo.17037240).
